# Expression Patterns of PACAP and PAC1R Genes and Anorexigenic Action of PACAP1 and PACAP2 in Zebrafish

**DOI:** 10.3389/fendo.2019.00227

**Published:** 2019-04-12

**Authors:** Tomoya Nakamachi, Ayano Tanigawa, Norifumi Konno, Seiji Shioda, Kouhei Matsuda

**Affiliations:** ^1^Laboratory of Regulatory Biology, Graduate School of Science and Engineering, University of Toyama, Toyama, Japan; ^2^Innovative Drug Discovery, Global Research Center for Innovative Life Science, Hoshi University, Tokyo, Japan

**Keywords:** feeding behavior, real-time PCR, tissue distribution, intracerebroventricular injection, PACAP receptor, genome duplication

## Abstract

Pituitary adenylate cyclase-activating polypeptide (PACAP) is a neuropeptide with potent suppressive effects on feeding behavior in rodents, chicken, and goldfish. Teleost fish express two PACAPs (PACAP1, encoded by the *adcyap1a* gene, and PACAP2, encoded by the *adcyap1b* gene) and two PACAP receptors (PAC1Rs; PAC1Ra, encoded by the *adcyap1r1a* gene, and PAC1Rb, encoded by the *adcyap1r1b* gene). However, the mRNA expression patterns of the two PACAPs and PAC1Rs, and the influence and relationship of the two PACAPs on feeding behavior in teleost fish remains unclear. Therefore, we first examined mRNA expression patterns of PACAP and PAC1R in tissue and brain. All PACAP and PAC1Rs mRNAs were dominantly expressed in the zebrafish brain. However, *adcyap1a* mRNA was also detected in the gut and testis. In the brain, *adcyap1b* and *adcyap1r1a* mRNA levels were greater than that of *adcyap1a* and *adcyap1r1b*, respectively. Moreover, *adcyap1b* and *adcyap1r1a* mRNA were dominantly expressed in telencephalon and diencephalon. The highest *adcyap1a* mRNA levels were detected in the brain stem and diencephalon, while the highest levels of *adcyap1r1b* were detected in the cerebellum. To clarify the relationship between PACAP and feeding behavior in the zebrafish, the effects of zebrafish (zf) PACAP1 or zfPACAP2 intracerebroventricular (ICV) injection were examined on food intake, and changes in PACAP mRNA levels were assessed against feeding status. Food intake was significantly decreased by ICV injection of zfPACAP1 (2 pmol/g body weight), zfPACAP2 (2 or 20 pmol/g body weight), or mammalian PACAP (2 or 20 pmol/g). Meanwhile, the PACAP injection group did not change locomotor activity. Real-time PCR showed *adcyap1* mRNA levels were significantly increased at 2 and 3 h after feeding compared with the pre-feeding level, but *adcyap1b, adcyap1r1a*, and *adcyap1r1b* mRNA levels did not change after feeding. These results suggest that the expression levels and distribution of duplicated PACAP and PAC1R genes are different in zebrafish, but the anorexigenic effects of PACAP are similar to those seen in other vertebrates.

## Introduction

Pituitary adenylate cyclase-activating polypeptide (PACAP) is a bioactive peptide that was originally isolated from the ovine hypothalamus as an activator of cAMP production in pituitary cells (1). It belongs to the vasoactive intestinal polypeptide (VIP)/secretin/glucagon superfamily, and its closest paralog is VIP. PACAP and VIP share three types of G protein-coupled receptors: PACAP-type 1 receptor (PAC1R), VPAC1 receptor, and VPAC2 receptor. The binding affinity of PACAP to PAC1R is ~1,000 times greater than that of VPAC receptors, suggesting that PAC1R is the dominant receptor for PACAP (2). PACAP and PAC1R are widely distributed in central nervous tissues and peripheral tissues in mammals (3, 4). Although PACAP is encoded by the *adcyap1* gene in tetrapods, teleost fish have two PACAPs (PACAP1, encoded by the *adcyap1a* gene and PACAP2, encoded by the *adcyap1b* gene) and two PACAP receptors (PAC1Rs; PAC1Ra, encoded by the *adcyap1r1a* gene, and PAC1Rb, encoded by the *adcyap1r1b* gene) by teleost-specific whole-genome duplication (5, 6). We previously reported the tissue distribution of PACAP2 and *adcyap1b* in zebrafish (7); however, the correlation between expression and distribution of the two PACAPs and PAC1Rs has not yet been clarified.

The amino acid sequence of PACAP is well-conserved in vertebrates, implying that it has important physiological functions for biological activities. Indeed, PACAP contributes a variety of physiological functions, such as neuroprotection (8, 9), retinal protection (3, 10), glial regulation (11–13), neural development (14, 15), immunomodulation (16, 17), exocrine secretion (18, 19), stress response (20–22), and affecting the memory and learning system (23, 24). PACAP also act as a neuromodulator, and its anorectic effects have been widely studied in vertebrates (25, 26). It was reported that intracerebroventricular (ICV) administration of frog PACAP suppressed food intake in goldfish (27). However, the influence of duplicated PACAP/PAC1R genes in fish on feeding behavior has not yet been clarified.

Therefore, the present study used real-time PCR to quantify the distribution and expression levels of the two PACAP and PAC1R genes expressed in adult zebrafish. Furthermore, the influence of administering zebrafish (zf) PACAP1, zfPACAP2, and mammalian PACAP (mPACAP) on food intake was investigated, and the expression patterns of PACAP and PAC1R genes after feeding were evaluated.

## Materials and Methods

### Animals and Housing

Adult short-fin strain zebrafish were purchased from a local commercial supplier in Toyama City. Fish were kept in 35-L housing tanks for at least 2 weeks under standard conditions (28 ± 1°C, 14 h light/10 h dark cycle) prior to experiments. Fish were fed twice per day with brine shrimp (*Artemia salina*) and an artificial diet (Hikari-labo series; Kyorin Co., Ltd., Himeji, Japan). All protocols were conducted in accordance with the University of Toyama guidelines for the care and use of animals.

### Reagents

Three types of PACAP were used for ICV injection. mPACAP38 was purchased from Peptide Institute Inc., Japan. zfPACAP1 (HSDGVFTDSYSRYRKQMAVKKYLATVLGKRYRQRYRSK-NH2) and zfPACAP2 (HSDGIFTDIYSRYRKQMAVKKYLAAVL-GRRYRQRVKNK-NH2) were synthesized by Toray Research Center Inc., Japan, with over 95% purity.

### Standard DNA Fragment Preparation for Quantitative Real-Time PCR

Standard DNA fragments were prepared for absolute quantification of each gene. Gene-specific primers for standard samples of the two PACAP genes, *adcyap1a* (NM_152885.2) and *adcyap1b* (NM_214715), and the two PAC1R genes, *adcyap1r1a* (NM_001013444.2) and *adcyap1r1b* (XM_677888.7), including the whole open reading frame region were designed using the Primer3Plus website (https://primer3plus.com/) (Table 1). Zebrafish were deeply anesthetized in ice-cold water, and the whole brain was removed. Total RNA was isolated from frozen tissues using TriPure isolation reagent (Sigma-Aldrich, St Louis, MO, USA) according to the manufacturer's instructions. cDNAs were synthesized using PerfectScript RT reagent kit using gDNA Eraser (TAKARA Bio, Otsu, Japan). PCR was performed using KOD FX Neo (Toyobo Co. Ltd, Tokyo Japan) with a Takara PCR Thermal Cycler Dice Standard (TAKARA Bio). The PCR step comprised 98°C for the first 2 min, followed by 40 cycles of 98°C for 10 s, 58°C for 30 s, and 68°C for 1 min. After agarose electrophoresis, target bands were cut out and DNA was isolated using NucleoSpin Gel and PCR Clean-up kit (TAKARA Bio). DNA concentration was quantified by Implen NanoPhotometer P-class (Implen GmbH, München, Germany) and the number of molecules of the standard sample for each gene was calculated.

**Table 1:** Oligonucleotides used as primers of PCR for standard template and quantitative PCR.

**Target**	**Sequence**	**Amplicon length**
**FOR STANDARD TEMPLATE DNA**		
adcyap 1a-Forward	AGCCTCCATTGGACAGCATC	589 bp
adcyap 1a-Reverse	ATTGATGGTTAGAGAGAGACGC	
adcyap 1b-Forward	AAGAGGTGCTGTGAGGAAGA	1,191 bp
adcyap 1b-Reverse	CGTGCCAGCTACTTTTCATC	
adcyap 1r1a-Forward	CACACGAACATACCTCCATCTG	1,621 bp
adcyap 1r1a Reverse	GCAGGTGAAGAGAGTGAGTTGA	
adcyap 1r1b-Forward	TATAATCGCTTCAGTGCTCCA	1,397 bp
adcyap 1r1b-Reverse	GTGTTTGGCCTTCAGTTTGTG	
**FOR REALTIME-PCR**		
adcyap 1a-Forward	AGCCTCCATTGGACAGCATC	149 bp
adcyap 1a-Reverse	CAGAGGCGAACACACATTGC	
adcyap 1b-Forward	TCTCACACAATCACAGCCGC	158 pb
adcyap 1b-Reverse	AGTCATCCCAATAGGCGTGC	
adcyap 1r1a-Forward	TCTGGTGGGTGATCAAGGGC	95 bp
adcyap 1r1a-Reverse	CTGAAGCTTCTGCACCAGGA	
adcyap 1r1b-Forward	TGATCCCAACAGTGAACCGG	84 bp
adcyap 1r1b-Reverse	ACTTGACCCAGTCTTGCAGG	

### Quantitative Real-Time PCR

Zebrafish were deeply anesthetized in ice-cold water. For tissue expression analysis, whole brain, eye, gill, heart, gut, kidney, liver, spleen, skin, muscle, and testes were isolated from male zebrafish, and ovaries were isolated from female zebrafish (*n* = 5 in each sample). For brain expression analysis, the whole brain was divided into six regions: telencephalon, optic tectum, diencephalon, cerebellum, brain stem, and spinal cord (*n* = 5 in each brain region). The tissues were immediately stored at −80°C until used. Total RNA extraction and cDNA synthesis were performed using the protocol described above. Gene-specific primers for real-time PCR were designed using the Primer3Plus website (Table 1). PCR was performed using TB Green Premix Ex Taq II reagent (TAKARA Bio) with a CFX Connect real-time PCR system (Bio-Rad Laboratories, Inc. Hercules, CA, USA). The PCR reaction was run at 95°C for the first 5 min, followed by 40 cycles at 95°C for 5 s, 60°C for 30 s, and 65°C for 5 s. A dissociation step from 60 to 95°C was performed at the end of the run. PCR was performed using cDNA from tissue and standard samples, and gene expression levels were quantified using a standard curve of Ct values from the standard samples.

### ICV Administration and Quantification of Food Intake

Female zebrafish (4 to 5 cm) were used for ICV administration because they are larger; therefore, ICV injection is easier in females compared with male zebrafish. ICV administration was performed as previously reported (28). Zebrafish were maintained under fasting conditions for 24 h in 3-L tanks individually prior to experiments. Zebrafish were anesthetized using 0.01% eugenol (Wako Pure Chemical Industries, Ltd, Osaka, Japan), and placed between a water-soaked sponge. A tiny hole was drilled in the skull on the third ventricle (between telencephalon and optic tectum) using a 25G needle (Terumo Co, Tokyo, Japan; outer diameter = 0.5 mm). Then, a 35G needle (React System, Osaka, Japan; outer diameter = 0.15 mm) attached to a Hamilton syringe was inserted into the third ventricle of the brain and test solution [2 or 20 pmol/g body weight (BW) of mPACAP, zfPACAP1, or zfPACAP2, 7–20 fish in each group] including 0.1% Evans blue dye was injected over a period of 2 min. The volume of the injected-solution is 0.5 μl/g BW. The doses of PACAP was determined with reference to previous paper which showed ICV administration of PACAP in goldfish (29). Saline-injected group was used as control (*n* = 26). The needle was removed, and the hole was filled using cyanoacrylate surgical glue. The zebrafish were then returned to the 3-L tank, and kept for 15 min for recovery from the anesthesia. After the behavior study, zebrafish were anesthetized with ice-cold water, and the brains removed. The brain was dissected in the ICV region, and positive stained by Evans blue in the interior of the cerebral ventricle indicated successful ICV administration. Success rate of ICV injection was ~50–70%. The brain was dissected after behavioral analysis and fish which did not observe Evans blue in the ventricle were excluded from the experimental group.

### Quantification of Food Intake

Brine shrimps were divided equally into 20 1.5-mL tubes, and the number of brine shrimps in three tubes were randomly selected and counted. After ICV administration, brine shrimps were placed in the tank and zebrafish were fed the brine shrimp *ad libitum* for 15 min. If most of the brine shrimps in the tank were consumed (approximately <20 brine shrimps), other tubes with brine shrimps were added. After the feeding period had ended, the zebrafish were immediately moved to another tank, and the remaining brine shrimps were filtered and counted. Food intake was calculated using the following formula:

(Average no. of brine shrimps in three tubes) × (No. of tubes fed) – (No. of remaining brine shrimps in the tank).

### Quantification of Locomotor Activity

After a 15-min recovery interval following ICV administration, zebrafish (10–13 fish in each group) were transferred to the white circle water tank, with a diameter of 26 cm, and 2 L of breeding water at 28°C. Behavior was monitored for 15 min using a 1/3 inch compact and high sensitivity camera (WAT-250D2, Watec Co., Ltd. Tokyo, Japan). The locomotor activity (total distance) was quantified using a video tracking system, ANY-maze (Stoeling Co., Wood Dale, IL).

### Changes in PACAP and PAC1R mRNAs Level After Feeding

Male zebrafish were used in this experiment to avoid the influence of the estrus cycle. Twenty zebrafish were transferred to a 40-L water tank, and acclimatized by feeding 2% weight of artificial diet showing above at 10:00 a.m. once a day for 5 days. Five zebrafish were randomly selected from the tank 30 min before, and 1, 2, and 3 h after feeding on day 5, and brains were isolated under ice-cooled anesthesia. The diencephalic brain including hypothalamic region, which is a known feeding center, was used for real-time PCR to quantify PACAP and PAC1R mRNA levels. Ten to fifteen fish were used in each group.

## Statistics

Experimental data are presented as the mean ± S.E. (*n* = sample size). Statistical analysis was performed by one-way ANOVA followed by the Dunnett test. *P* < 0.05 was considered statistically significant.

## Results

### Expression Pattern of zfPACAP and zfPAC1R mRNA in Zebrafish Tissues and Brain Regions

**Figure 1:**
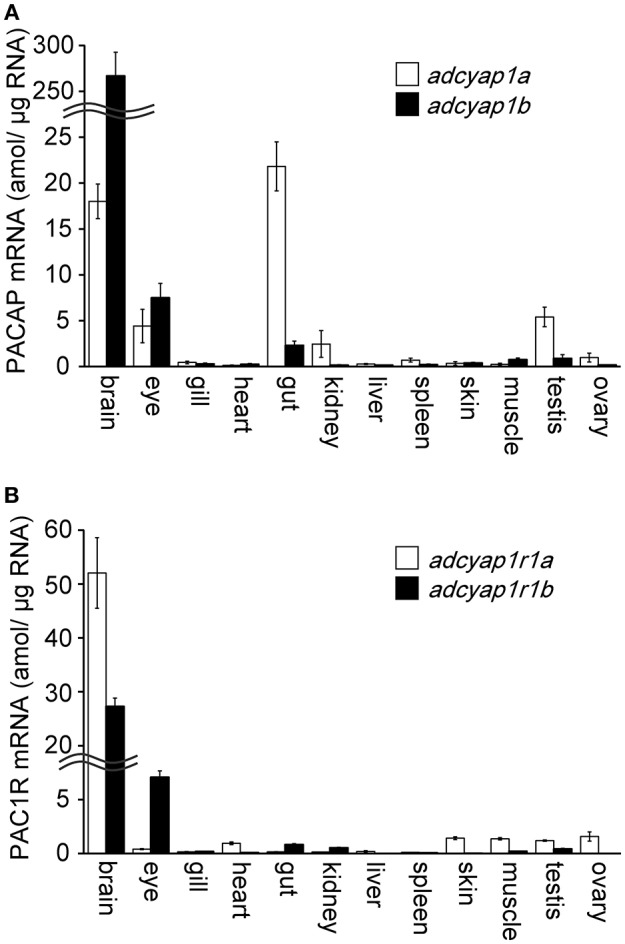
Tissue distribution of zebrafish PACAP and PAC1R mRNA. Distribution and expression of the PACAP mRNA, *adcyap1a* and *adcyap1b* genes **(A)**, PAC1R mRNA, *adcyap1r1a* and *adcyap1r1b* genes **(B)** were determined using absolute quantitative real-time PCR. Double wavy line indicates a break in the axis. *n* = 5 for each tissue.

Using real-time PCR, the highest levels of *adcyap1a* and *adcyap1b* mRNA were detected in the brain (Figure 1A). Levels of *adcyap1b* mRNA were almost 15 times higher than those of *adcyap1a* mRNA in the brain. *adcyap1b* mRNA was dominantly expressed in the brain and was also expressed at low levels in the eye. Additionally, adcyap1a mRNA levels in the gut were slightly similar to those in the brain, and low levels were detected in the testis and eye. Measurement of PAC1R mRNA revealed the highest levels of *adcyap1r1a* and *adcyap1r1b* mRNA were detected in the brain (Figure 1B). Furthermore, *adcyap1r1a* mRNA levels were almost twice as high than *adcyap1r1b* mRNA levels in the brain. *adcyap1r1b* mRNA was slightly expressed in the eye. However, *adcyap1r1a* and *adcyap1r1b* mRNAs were expressed at very low levels in the peripheral tissues.

**Figure 2:**
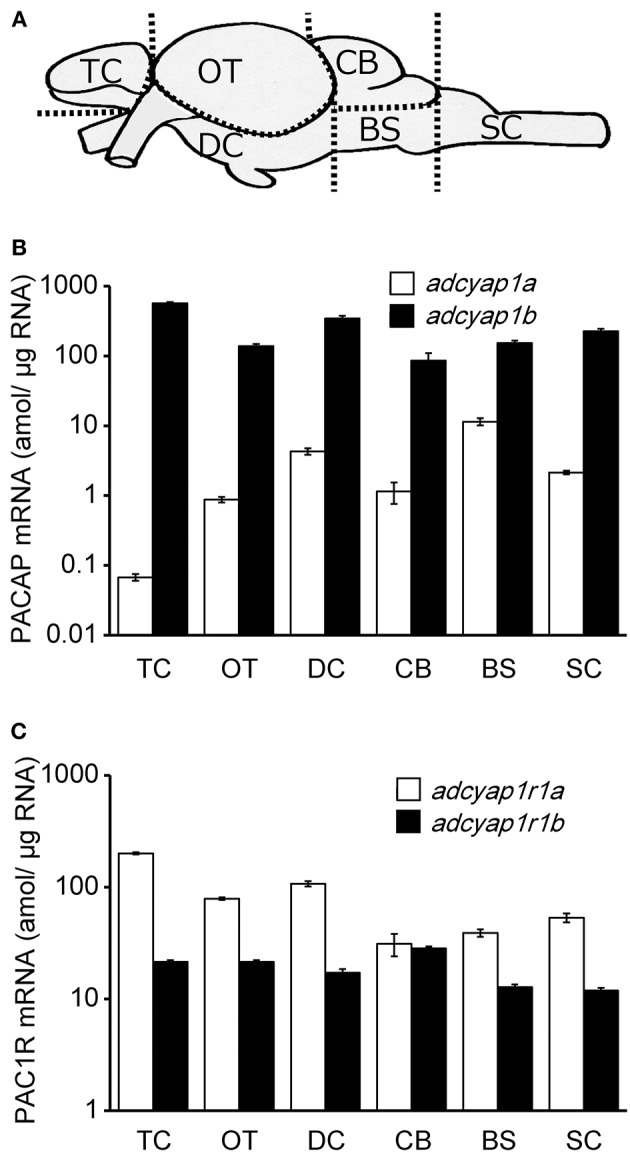
Brain distribution of zebrafish PACAP and PAC1R mRNA. Schematic diagram of brain region in zebrafish was shown in **(A)**. Distribution and expression of PACAP mRNA, *adcyap1a* and *adcyap1b* genes **(B)**, PAC1R mRNA, *adcyap1r1a* and *adcyap1r1b* genes **(C)** were determined using absolute quantitative real-time PCR. The *y*-axis is displayed on a logarithmic scale. *n* = 5 in each brain region. Abbreviations: TC, telencephalon; OT, optic tectum; DC, diencephalon; CB, cerebellum; BS, brain stem; SC, spinal cord.

Subsequently, the brain was divided into six regions, namely, telencephalic region, optic tectum, diencephalic region, cerebellum, brain stem, and spinal cord (Figure 2A), and PACAPs and PAC1R mRNA was measured in each brain region. In all six brain regions, *adcyap1b* mRNA levels were greater than those of *adcyap1a* (Figure 2B). The highest levels of *adcyap1a* and *adcyap1b* mRNAs were detected in the brain stem and telencephalon, respectively. Both PACAP mRNAs were expressed at relatively higher levels in the diencephalon. Measurement of PAC1R mRNA showed higher levels of *adcyap1r1a* mRNA levels compared with *adcyap1r1b* in all six brain regions (Figure 2C). High levels of *adcyap1r1a* mRNA were detected in the telencephalon and diencephalon, and the highest levels of *adcyap1r1b* mRNA were detected in the cerebellum.

### Effects of PACAP ICV Injection on Feeding Behavior and Locomotor Activity

**Figure 3:**
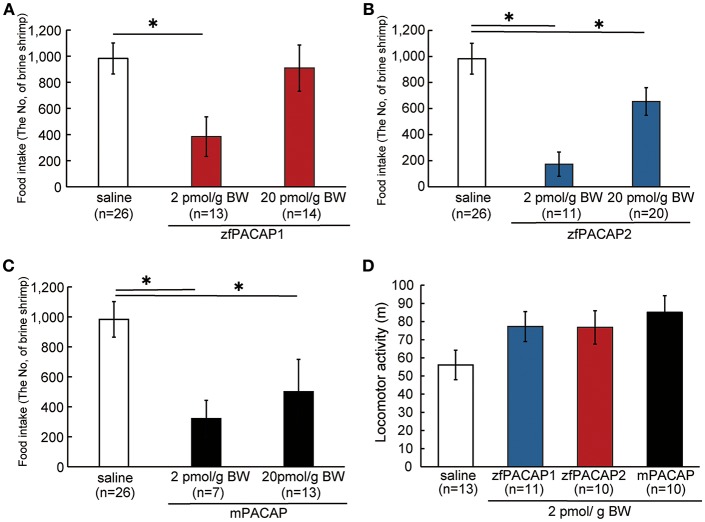
Food consumption and locomotor activity after ICV injection of PACAP. **(A–C)** Food intake was measured for a 15-min feeding period after ICV injection of PACAP. **(D)** Locomotor activity was measured in a white circle water tank for 15 min after ICV injection. ^*^P < 0.05.

To determine the effects of zebrafish PACAPs on feeding behavior, feeding behavior and locomotor activity were measured after ICV administration of mPACAP, zfPACAP1, and zfPACAP2. zfPACAP1 injection at 2 pmol/g significantly suppressed food intake for 15 min after injection, but not at 20 pmol/g (Figure 3A). zfPACAP2 and mPACAP injection at 2 and 20 pmol/g significantly suppressed food intake (Figures 3B,C). At 2 pmol/g, all three PACAP injection groups tended to increase their locomotor activity in a circular tank, but this was not significantly different (Figure 3D).

### Changes in PACAP and PACAP Receptor mRNA Before and After Feeding

**Figure 4:**
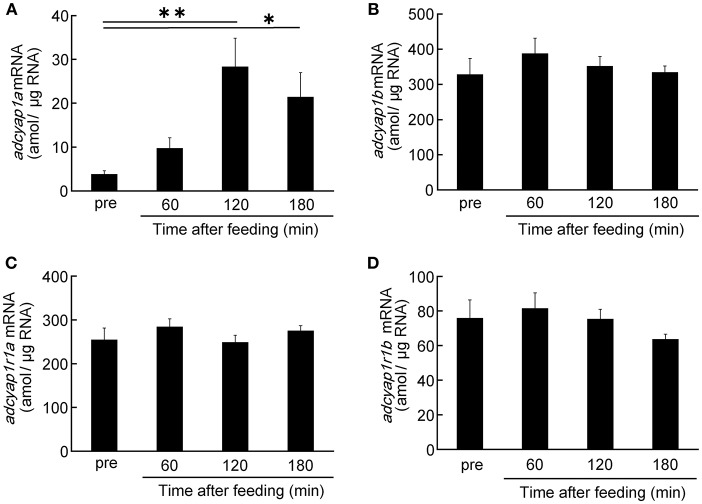
Time-course changes of PACAP and PAC1R mRNAs level after feeding. Expression levels of PACAP mRNA, *adcyap1a* **(A)** and *adcyap1b* **(B)** genes, PAC1R mRNA, *adcyap1r1a* **(C)** and *adcyap1r1b* **(D)** genes were determined using absolute quantitative real-time PCR. The number of samples in the pre, 60, 120, and 180 min groups were 13, 15, 15, and 10, respectively. ^*^P < 0.05, ^**^P < 0.01.

PACAP and PAC1R mRNA levels in the diencephalic region were quantified before and after feeding. *adcyap1a* mRNA levels tended to increase at 60 min after feeding, and peaked significantly at 120 and 180 min (Figure 4A). At the peak response at 120 min, *adcyap1a* mRNA levels were 7.5 times higher than the pre-feeding levels. While, mRNA levels of *adcyap1b, adcyap1r1a*, and *adcyap1r1b* did not change significantly until 180 min after feeding (Figures 4B–D).

## Discussion

### Tissue Expression and Distribution of Duplicated zfPACAP and zfPAC1R mRNA

The distribution and tissue expression of *adcyap1b* mRNA quantified by relative quantification real-time PCR was described previously (7); however, a comparison of the distribution and expression of the duplicated PACAP or PAC1R mRNA was not studied. Therefore, the present study used absolute quantified real-time PCR for this comparison. A comparison of expression levels in the brain showed levels of *adcyap1b* were around 15 times higher than those of *adcyap1a*, and levels of *adcyap1r1a* were around 2-fold higher than those of *adcyap1r1b*. These data indicate that duplicated zfPACAP and zfPAC1R have very different expression levels in the central nervous system. In the brain expression experiment, *adcyap1b* levels were higher than those of *adcyap1a* in all of the brain regions. While in the telencephalon, *adcyap1b* expression was maximal, but *adcyap1a* expression was lowest. This result is in agreement with previous results showing that *adcyap1b* mRNA and PACAP2 immunoreactivity are highly expressed in the telencephalon (7). Measurements of the PACAP receptor showed that *adcyaplr1a* mRNA was highly expressed in the telencephalon. These results indicate that the brain localization of duplicated PACAP and PAC1R genes is also different, implying that duplicated zfPACAP and zfPAC1R may be responsible for different physiological effects. The telencephalon of fish is thought to closely corresponds to the limbic system of the mammalian hippocampus, amygdala, and cerebral cortex (30). Indeed, PACAP and PAC1R are expressed in the hippocampus and amygdala in rats (4), and PACAP ICV administration to rats improved passive avoidance learning, suggesting that PACAP is involved in the memory and learning system (24). These reports indicate that *adcyap1b* and *adcyap1r1a* may be involved in the memory and learning system in zebrafish.

In the peripheral tissues, *adcyap1a* was expressed at high levels, and was most highly expressed in the intestinal tract. It was reported that PACAP acts on intestinal smooth muscle relaxation in mammals (31). In fish, PACAP-like immunopositive cells and nerve fibers were observed in the myenteric plexus and smooth muscle in the rectum, and PACAP relaxed smooth muscle in the stargazer (32). PACAP mRNA was detected in the stomach and intestine in largemouth bass (33). Therefore, PACAP may have smooth muscle relaxant actions in the intestinal tract in zebrafish. However, in the present study, despite the high expression of *adcyap1a* in the intestinal tissue, expression of *adcyap1r1a* and *adcyap1r1b* of zfPAC1R genes in the intestinal tract was only detected at very low levels. PACAP has the ability to bind to three receptors: PAC1R (with highest affinity), and two VIP receptors, VPAC1R and VPAC2R (2). PACAP may act in intestinal tissues via VPACR. To verify this, the expression and distribution of VPAC1R and VPAC2R in zebrafish intestinal tissues needs to be clarified.

Various pathological condition models have been developed for zebrafish. It has been reported that administration of PACAP could inhibit adriamycin-induced kidney damage and hydrogen peroxide-induced damage of sensory hair cells in zebrafish (34, 35). The results of the distribution of PACAP and PACAP receptors in the zebrafish obtained in this study are also important knowledge for the pathophysiological study of PACAP in zebrafish.

### Effects of zfPACAP1, zfPACAP2, or mPACAP ICV Administration on Food Intake and Locomotor Activity

The anorexigenic effects of PACAP have mainly been studied using rodent models. Local administration of PACAP into nuclei, such as the paraventricular nucleus (36), ventromedial nucleus (37), amygdala central nucleus (38), and the nucleus accumbens (39) reduced food intake, and PACAP exhibited anorexigenic activity via PAC1R (36). In non-mammalian species, ICV administration of human PACAP in chicks (40, 41) and frog PACAP in goldfish (27) reduced food intake. However, these experiments were conducted in PACAP from different species, and there are no reports on whether same species PACAP has truly anorexigenic effect in non-mammals. The present study revealed that ICV administration of both zfPACAPs suppressed food intake without affecting locomotor activity. This result indicates that zfPACAP has potent anorexigenic effects in the brain. PACAP1 and PACAP2 share 82% homology between their amino acid sequences (7). It seems that the 18% of the sequence that differs does not significantly influence the anorexigenic effect.

ICV administration of mPACAP also reduced food intake. This suggests that mPACAP has an affinity to zfPAC1R, and that zfPAC1R has similar ligand selectivity to mPAC1R. In fish, it is known that orexigenic peptides and anorexigenic peptides, which are mainly expressed in the hypothalamus, are involved in appetite regulation, and PACAP is considered to be one of the anorexigenic peptide among them (29, 42). In rats, PACAP increased expression levels of proopiomelanocortin (POMC) by activating the adenylate cyclase/cAMP/protein kinase A pathway via PAC1R, consequently demonstrating its anorexigenic action by secreting the anorexigenic hormone, alpha melanocyte stimulating hormone (α-MSH), induced by POMC (43, 44). On the other hand, PACAP exerts anorexigenic effects via corticotropin-releasing hormone (CRH) in goldfish (45). It is possible that the anorexigenic pathway in fish may differ from that of mammals. On the other hand, administration of PACAP to juvenile tilapia has been reported to enhance growth and feeding (46). This suggests that the effect of PACAP may vary depending on the route of administration, fish species, or stage of development. Further studies are required to determine whether the anorexigenic action of PACAP in zebrafish is via a Gs pathway, and other appetite-regulating hormones, such as α-MSH and CRH.

The PACAP ICV administration experiment showed a significant suppressive effect on food intake only at low concentrations (2 pmol/g BW) of PACAP. The neuroprotective action of PACAP showing rat retinal ganglion cell death (47), mouse retinal injury (48), spinal cord injury (49), and exocrine stimulation in mouse tear secretion (18) demonstrated a bell-shaped dose response, in other words, the action of PACAP peaks and decreases at high concentrations. Although the details remain unknown, there may be a relationship between the response difference of PAC1R with high affinity and VPAC1R and VPAC2R with relatively low affinity. Two VPAC1R genes and one VPAC2R gene were identified in the zebrafish genome on the genome database. Future investigations into the affinity of zfPAC1Rs and zfVPACRs for zfPACAP are required.

### Changes in zfPACAP and zfPAC1R mRNAs Levels and Feeding Conditions

There are still few reports on changes in the expression level of PACAP in response to feeding states. Kiss et al. reported that PACAP concentration was elevated in the rat brain at 12 h after food deprivation, and in the chicken brain at 36 h after food deprivation (50). PACAP and PAC1R mRNA expression was increased in overfeeding goldfish (51). Conversely, PACAP mRNA level was decreased in 4 days fasted largemouth bass (33). From these reports, it has been clarified that PACAP shows an expression pattern as an anorexigenic factor in fish, and our results are also consistent with this. However, it is still unclear why the expression change in the feeding states of PACAP differs between tetrapods and fish. This difference seems to have occurred due to evolutionally changes in vertebrate, or difference of the measurement method of PACAP in the peptide level or mRNA level.

In the present study, only *adcyap1a* mRNA was significantly increased at 2 and 3 h after feeding. Although ICV administration of both zfPACAP1 and zfPACAP2 suppressed food intake, it is noteworthy that only *adcyap1a* responded to the feeding state. This result suggests that zfPACAP1 is mainly involved in suppression of food intake after feeding. We previously showed that zfPACAP2 immunoreactivity was localized in the hypothalamus (7); however, the brain localization of zfPACAP1 remains unknown. In future, it will be necessary to prepare a specific antibody against zfPACAP1 and identify the localization of zfPACAP1 in the hypothalamus. Furthermore, the tissue distribution of zfPACAP mRNA and zfPAC1R mRNA should be analyzed using *in situ* hybridization.

On the other hand, the problem with the protocol in this paper is that the experimental fish was continuously sampled from the same stock tank. Therefore, the remaining fish may have been under the stress. In other words, elevation of adcyap1a mRNA after feeding may have been caused by a stress response. Actually, chronic stress increases endogenous PACAP expression in the hypothalamic nucleus, and PACAP can stimulate CRH production and secretion in rodents (21). It is necessary to review and upgrade the protocol, and clarify the relationship between PACAP and food intake under less stressful conditions.

In conclusion, expression levels and the distribution of zfPACAPs and zfPAC1Rs differ in zebrafish. There was almost no difference in the anorexigenic effects of zfPACAP1 and zfPACAP2 by ICV administration. These results suggest that PACAP act as an anorexigenic feeding regulator in the brain, and the anorexigenic effect of PACAP is preserved in vertebrates. Molecular species of the VIP/secretin/glucagon superfamily are thought to have been formed by repeating exon and gene duplication from PACAP-type ancestral genes during evolution (52). It is possible that duplicated zfPACAPs have begun to evolve into another paralogous gene by altering its distribution and responses. Functional analysis of zfPACAP and zfPAC1R, further physiological experiments, and detailed tissue distribution observation will clarify this hypothesis.

## Ethics Statement

All experimental procedures were conducted in accordance with the institutional guidelines of the University of Toyama for the care and use of laboratory animals. However, it was not necessary to receive permission for fish experiment in the University of Toyama. No endangered animal species were involved in the study.

## Author Contributions

TN and AT designed and performed the experiments. NK, SS, and KM were involved in planning and supervising the work. TN wrote the manuscript with support from NK, SS, and KM.

### Conflict of Interest Statement

The authors declare that the research was conducted in the absence of any commercial or financial relationships that could be construed as a potential conflict of interest.

## References

[1] Hirabayashi T Nakamachi T Shioda S. Discovery of PACAP and its receptors in the brain. J Headache Pain. (2018) 19:28. 10.1186/s10194-018-0855-1 29619773 PMC5884755

[2] Harmar AJ Fahrenkrug J Gozes I Laburthe M May V Pisegna JR. Pharmacology and functions of receptors for vasoactive intestinal peptide and pituitary adenylate cyclase-activating polypeptide: IUPHAR Review 1. Br J Pharmacol. (2012) 166:4–17. 10.1111/j.1476-5381.2012.01871.x 22289055 PMC3415633

[3] Nakamachi T Matkovits A Seki T Shioda S. Distribution and protective function of pituitary adenylate cyclase-activating polypeptide in the retina. Front Endocrinol (Lausanne). (2012) 3:145. 10.3389/fendo.2012.00145 23189073 PMC3504973

[4] Vaudry D Falluel-Morel A Bourgault S Basille M Burel D Wurtz O. Pituitary adenylate cyclase-activating polypeptide and its receptors: 20 years after the discovery. Pharmacol Rev. (2009) 61:283–357. 10.1124/pr.109.001370 19805477

[5] Cardoso JCR Félix RC Martins RST Trindade M Fonseca VG Fuentes J. PACAP system evolution and its role in melanophore function in teleost fish skin. Mol Cell Endocrinol. (2015) 411:130–45. 10.1016/j.mce.2015.04.020 25933704

[6] On JS Chow BK Molecular evolution of pituitary adenylate cyclase-activating polypeptide subfamily and cognate receptor subfamily In: Reglodi D Tamas A, editors. Current Topics in Neurotoxicity. Pituitary Adenylate Cyclase Activating Polypeptide – PACAP. Berlin: Springer Nature (2016). p. 3–17.

[7] Nakamachi T Kamata E Tanigawa A Konno N Shioda S Matsuda K. Distribution of pituitary adenylate cyclase-activating polypeptide 2 in zebrafish brain. Peptides. (2018) 103:40–7. 10.1016/j.peptides.2018.03.006 29535004

[8] Ohtaki H Nakamachi T Dohi K Shioda S. Role of PACAP in ischemic neural death. J Mol Neurosci. (2008) 36:16–25. 10.1007/s12031-008-9077-3 18483879

[9] Shioda S Nakamachi T. PACAP as a neuroprotective factor in ischemic neuronal injuries. Peptides. (2015) 72:202–7. 10.1016/j.peptides.2015.08.006 26275482

[10] Shioda S Takenoya F Wada N Hirabayashi T Seki T Nakamachi T. Pleiotropic and retinoprotective functions of PACAP. Anat Sci Int. (2016) 91:313–24. 10.1007/s12565-016-0351-0 27324639

[11] Masmoudi-Kouki O Gandolfo P Castel H Leprince J Fournier A Dejda A. Role of PACAP and VIP in astroglial functions. Peptides. (2007) 28:1753–60. 10.1016/j.peptides.2007.05.015 17655978

[12] Nakamachi T Nakamura K Oshida K Kagami N Mori H Watanabe J. Pituitary adenylate cyclase-activating polypeptide (PACAP) stimulates proliferation of reactive astrocytes *in vitro*. J Mol Neurosci. (2011b) 43:16–21. 10.1007/s12031-010-9404-3 20574684

[13] Nakamachi T Farkas J Watanabe J Ohtaki H Dohi K Arata S. Role of PACAP in neural stem/progenitor cell and astrocyte: from neural development to neural repair. Curr Pharm Des. (2011a) 17:973–84. 10.2174/138161211795589346 21524256

[14] Shioda S Ohtaki H Nakamachi T Dohi K Watanabe J Nakajo S. Pleiotropic functions of PACAP in the CNS: Neuroprotection and neurodevelopment. Ann N Y Acad Sci. (2006) 1070:550–60. 10.1196/annals.1317.080 16888224

[15] Watanabe J Nakamachi T Matsuno R Hayashi D Nakamura M Kikuyama S. Localization, characterization and function of pituitary adenylate cyclase-activating polypeptide during brain development. Peptides. (2007) 28:1713–9. 10.1016/j.peptides.2007.06.029 17719696

[16] Wada Y Nakamachi T Endo K Seki T Ohtaki H Tsuchikawa D. PACAP attenuates NMDA-induced retinal damage in association with modulation of the microglia/macrophage status into an acquired deactivation subtype. J Mol Neurosci. (2013) 51:493–502. 10.1007/s12031-013-0017-5 23720065

[17] Waschek JA. VIP and PACAP: neuropeptide modulators of CNS inflammation, injury, and repair. Br J Pharmacol. (2013) 169:512–23 10.1111/bph.12181 23517078 PMC3682700

[18] Nakamachi T Ohtaki H Seki T Yofu S Kagami N Hashimoto H. PACAP suppresses dry eye signs by stimulating tear secretion. Nat Commun. (2016) 7:12034. 10.1038/ncomms12034 27345595 PMC4931240

[19] Sasaki S Watanabe J Ohtaki H Matsumoto M Murai N Nakamachi T. Pituitary adenylate cyclase-activating polypeptide promotes eccrine gland sweat secretion. Br J Dermatol. (2017) 176:413–22. 10.1111/bjd.14885 27453364

[20] Hashimoto H Shintani N Tanida M Hayata A Hashimoto R Baba A. PACAP is implicated in the stress axes. Curr Pharm Des. (2011) 17:985–9. 10.2174/138161211795589382 21524255 PMC3179129

[21] Hammack SE May V. Pituitary adenylate cyclase activating polypeptide in stress-related disorders: data convergence from animal and human studies. Biol Psychiatry. (2015) 78:167–77. 10.1016/j.biopsych.2014.12.003 25636177 PMC4461555

[22] King SB Toufexis DJ Hammack SE. Pituitary adenylate cyclase activating polypeptide (PACAP), stress, and sex hormones. Stress. (2017) 20:465–75. 10.1080/10253890.2017.1336535 28610473 PMC6724739

[23] Adamik A Telegdy G. Effects of pituitary adenylate cyclase polypeptide (PACAP) on extinction of active avoidance learning in rats: involvement of neurotransmitters. Regul Pept. (2005) 127:55–62. 10.1016/j.regpep.2004.10.015 15680470

[24] Sacchetti B Lorenzini CA Baldi E Bucherelli C Roberto M Tassoni G. Pituitary adenylate cyclase-activating polypeptide hormone (PACAP) at very low dosages improves memory in the rat. Neurobiol Learn Mem. (2001) 76:1–6. 10.1006/nlme.2001.4014 11525248

[25] Matsuda K Maruyama K. Regulation of feeding behavior by pituitary adenylate cyclase-activating polypeptide (PACAP) and vasoactive intestinal polypeptide (VIP) in vertebrates. Peptides. (2007) 28:1761–6. 10.1016/j.peptides.2007.03.007 17466413

[26] Sekar R Wang L Chow BKC. Central control of feeding behavior by the secretin, pacap, and glucagon family of peptides. Front Endocrinol (Lausanne). (2017) 8:18. 10.3389/fendo.2017.00018 28223965 PMC5293785

[27] Matsuda K Maruyama K Nakamachi T Miura T Uchiyama M Shioda S. Inhibitory effects of pituitary adenylate cyclase-activating polypeptide (PACAP) and vasoactive intestinal peptide (VIP) on food intake in the goldfish, *Carassius auratus*. Peptides. (2005) 26:1611–6. 10.1016/j.peptides.2005.02.022 16112400

[28] Nishiguchi R Azuma M Yokobori E Uchiyama M Matsuda K. Gonadotropin-releasing hormone 2 suppresses food intake in the zebrafish, *Danio rerio*. Front Endocrinol (Lausanne). (2012) 3:122. 10.3389/fendo.2012.00122 23087673 PMC3473230

[29] Matsuda K Maruyama K Nakamachi T Miura T Shioda S. Effects of pituitary adenylate cyclase-activating polypeptide and vasoactive intestinal polypeptide on food intake and locomotor activity in the goldfish, *Carassius auratus*. Ann N Y Acad Sci. (2006) 1070:417–21. 10.1196/annals.1317.054 16888202

[30] Rodríguez-Expósito B Gómez A Martín-Monzón I Reiriz M Rodríguez F Salas C. Goldfish hippocampal pallium is essential to associate temporally discontiguous events. Neurobiol Learn Mem. (2017) 139:128–34. 10.1016/j.nlm.2017.01.002 28065713

[31] Al-Qudah M Alkahtani R Akbarali HI Murthy KS Grider JR. Stimulation of synthesis and release of brain-derived neurotropic factor from intestinal smooth muscle cells by substance P and pituitary adenylate cyclase-activating peptide. Neurogastroenterol Motil. (2015) 27:1162–74. 10.1111/nmo.12604 26088546 PMC4520799

[32] Matsuda K Kashimoto K Higuchi T Yoshida T Uchiyama M Shioda S Presence of pituitary adenylate cyclase-activating polypeptide (PACAP) and its relaxant activity in the rectum of a teleost, the stargazer, *Uranoscopus japonicus*. Peptides. (2000) 21:821–7. 10.1016/S0196-9781(00)00215-1 10959004

[33] ShengjieL Linqiang H Junjie B Dongmei M Yingchun Q Jiajia F Cloning, tissue distribution and effects of fasting on pituitary adenylate cyclase-activating polypeptide in largemouth bass. Chin J Oceanol Limn. (2015) 33:328–38. 10.1007/s00343-015-4081-2

[34] Eneman B Elmonem MA van den Heuvel LP Khodaparast L Khodaparast L van Geet C. Pituitary adenylate cyclase-activating polypeptide (PACAP) in zebrafish models of nephrotic syndrome. PLoS ONE. (2017) 12:e0182100. 10.1371/journal.pone.0182100 28759637 PMC5536324

[35] Kasica N Podlasz P Sundvik M Tamas A Reglodi D Kaleczyc J. Protective effects of pituitary adenylate cyclase-activating polypeptide (PACAP) against oxidative stress in zebrafish hair cells. Neurotox Res. (2016) 30:633–47. 10.1007/s12640-016-9659-8 27557978 PMC5047952

[36] Resch JM Maunze B Gerhardt AK Magnuson SK Phillips KA Choi S. Intrahypothalamic pituitary adenylate cyclase-activating polypeptide regulates energy balance via site-specific actions on feeding and metabolism. Am J Physiol Metab. (2013) 305:E1452–63. 10.1152/ajpendo.00293.2013 24148346 PMC3882380

[37] Resch JM Boisvert JP Hourigan AE Mueller CR Yi SS Choi S. Stimulation of the hypothalamic ventromedial nuclei by pituitary adenylate cyclase-activating polypeptide induces hypophagia and thermogenesis. Am J Physiol Integr Comp Physiol. (2011) 301:R1625–34. 10.1152/ajpregu.00334.2011 21957159 PMC3233848

[38] Iemolo A Ferragud A Cottone P Sabino V. Pituitary adenylate cyclase-activating peptide in the central amygdala causes anorexia and body weight loss via the melanocortin and the TrkB systems. Neuropsychopharmacology. (2015) 40:1846–55. 10.1038/npp.2015.34 25649277 PMC4839508

[39] Hurley MM Maunze B Block ME Frenkel MM Reilly MJ Kim E. Pituitary adenylate-cyclase activating polypeptide regulates hunger- and palatability-induced binge eating. Front Neurosci. (2016) 10:383. 10.3389/fnins.2016.00383 27597817 PMC4993128

[40] Tachibana T Saito ES Takahashi H Saito S Tomonaga S Boswell T. Anorexigenic effects of pituitary adenylate cyclase-activating polypeptide and vasoactive intestinal peptide in the chick brain are mediated by corticotrophin-releasing factor. Regul Pept. (2004) 120:99–105. 10.1016/j.regpep.2004.02.016 15177926

[41] Tachibana T Saito S Tomonaga S Takagi T Saito E-S Boswell T. Intracerebroventricular injection of vasoactive intestinal peptide and pituitary adenylate cyclase-activating polypeptide inhibits feeding in chicks. Neurosci Lett. (2003) 339:203–6. 10.1016/S0304-3940(03)00017-X 12633888

[42] Volkoff H. The neuroendocrine regulation of food intake in fish: a review of current knowledge. Front Neurosci. (2016) 10:540. 10.3389/fnins.2016.00540 27965528 PMC5126056

[43] Aoki Y Iwasaki Y Katahira M Oiso Y Saito H Regulation of the rat proopiomelanocortin gene expression in AtT-20 cells. II: Effects of the pituitary adenylate cyclase-activating polypeptide and vasoactive intestinal polypeptide. Endocrinology. (1997) 138:1930–4. 10.1210/endo.138.5.5116 9112389

[44] Mounien L Do Rego J-C Bizet P Boutelet I Gourcerol G Fournier A. Pituitary adenylate cyclase-activating polypeptide inhibits food intake in mice through activation of the hypothalamic melanocortin system. Neuropsychopharmacology. (2009) 34:424–35. 10.1038/npp.2008.73 18536705

[45] Matsuda K Kojima K Shimakura SI Wada K Maruyama K Uchiyama M. Corticotropin-releasing hormone mediates α-melanocyte-stimulating hormone-induced anorexigenic action in goldfish. Peptides. (2008) 29:1930–6. 10.1016/j.peptides.2008.06.028 18656512

[46] Lugo JM Oliva A Morales A Reyes O Garay HE Herrera F. The biological role of pituitary adenylate cyclase-activating polypeptide (PACAP) in growth and feeding behavior in juvenile fish. J Pept Sci. (2010) 16:633–43. 10.1002/psc.1275 20853308

[47] Seki T Itoh H Nakamachi T Shioda S. Suppression of ganglion cell death by PACAP following optic nerve transection in the rat. J Mol Neurosci. (2008) 36:57–60. 10.1007/s12031-008-9091-5 18642101

[48] Endo K Nakamachi T Seki T Kagami N Wada Y Nakamura K. Neuroprotective effect of PACAP against NMDA-induced retinal damage in the mouse. J Mol Neurosci. (2011) 43:22–9. 10.1007/s12031-010-9434-x 20703829

[49] Tsuchida M Nakamachi T Sugiyama K Tsuchikawa D Watanabe J Hori M. PACAP stimulates functional recovery after spinal cord injury through axonal regeneration. J Mol Neurosci. (2014) 54:380–7. 10.1007/s12031-014-0338-z 25074795

[50] Kiss P Reglodi D Tamás A Lubics A Lengvári I Józsa R. Changes of PACAP levels in the brain show gender differences following short-term water and food deprivation. Gen Comp Endocrinol. (2007) 152:225–30. 10.1016/j.ygcen.2006.12.012 17286974

[51] Matsuda K Maruyama K Miura T Uchiyama M Shioda S. Anorexigenic action of pituitary adenylate cyclase-activating polypeptide (PACAP) in the goldfish: feeding-induced changes in the expression of mRNAs for PACAP and its receptors in the brain, and locomotor response to central injection. Neurosci Lett. (2005) 386:9–13. 10.1016/j.neulet.2005.05.053 15975713

[52] Sherwood NM Krueckl SL McRory JE The origin and function of the pituitary adenylate cyclase-activating polypeptide (PACAP)/glucagon superfamily 1. Endocr Rev. (2000) 21:619–70. 10.1210/er.21.6.619 11133067

